# Efficacy of high-dose versus low-dose vitamin D supplementation on serum levels of inflammatory factors and mortality rate in severe traumatic brain injury patients: study protocol for a randomized placebo-controlled trial

**DOI:** 10.1186/s13063-020-04622-6

**Published:** 2020-07-29

**Authors:** Seyed Mostafa Arabi, Alireza Sedaghat, Mohammad Reza Ehsaei, Mohammad Safarian, Golnaz Ranjbar, Hamid Rezaee, Reza Rezvani, Hamed Tabesh, Abdolreza Norouzy

**Affiliations:** 1 Student Research Committee, Faculty of Medicine, Mashhad University of Medical Sciences, Mashhad, Iran; 2 Cardiac Anesthesia Research Center, Mashhad University of Medical Sciences, Mashhad, Iran; 3 Department of Neurosurgery, Shahid Kamiab Hospital, Faculty of Medicine, Mashhad University of Medical Sciences, Mashhad, Iran; 4 Metabolic Syndrome Research Center, Mashhad University of Medical Sciences, Mashhad, Iran; 5 Department of Medical Informatics, Faculty of Medicine, Mashhad University of Medical Sciences, Mashhad, Iran

**Keywords:** Traumatic brain injury, Vitamin D, Inflammation, Mortality

## Abstract

**Background:**

Traumatic brain injury (TBI) is the most common trauma worldwide and is a leading cause of injury-related death and disability. Inflammation is initiated as a result of the TBI, which is in association with severity of illness and mortality in brain trauma patients, especially in subdural hemorrhage and epidural hemorrhage cases. A high percentage of adults admitted to the intensive care unit with TBI are diagnosed with vitamin D deficiency; this deficiency may induce impaired immune responses and increase the risk of infections. Vitamin D intervention has been shown to modulate pro- and anti-inflammatory cytokines in non-critically ill patients, but to date, there is no substantial data on the effectiveness of vitamin D for the improvement of immune function in traumatic brain injury patients.

**Methods/design:**

A randomized clinical trial (RCT) will be performed on 74 Iranian adults 18–65 years old with brain trauma and will be treated daily with vitamin D supplements (100,000 IU oral drop) or a similar placebo (1000 IU) for 5 days.

**Discussion:**

If this randomized clinical trial demonstrates reductions in inflammatory cytokines, it would provide evidence for a multicenter clinical trial to evaluate the efficacy of vitamin D supplementation in neurocritically ill patients. Since vitamin D supplements are inexpensive and safe, this clinical trial could have the potential to improve clinical outcomes in traumatic brain injury patients through reduction of inflammation and infection-associated morbidity and mortality rates.

**Trial registration:**

Iranian Registry of Clinical Trials, IRCT20180619040151N3. Registered on 10 August 2019.

## Background

Vitamin D is an essential hormone for calcium homeostasis and its intestinal absorption. It also has an important role in several neuromuscular activities and metabolic responses [1]. Recent studies indicated that the active form of vitamin D (calcitriol) may have a crucial function in modulating immune responses to inflammatory conditions and infectious diseases [2, 3]. According to previous data, vitamin D deficiency has been associated with a high incidence of adverse events and mortality in critically ill patients [4]. Vitamin D can induce production of anti-inflammatory cytokines and some antimicrobial proteins such as cathelicidin by macrophages and neutrophils [3]. Antimicrobial proteins are expressed significantly in the respiratory epithelium and other organs, and they could reduce risk of infections in integumentary barrier sites [5, 6]. Notably, infectious diseases such as pneumonia and sepsis are common in traumatic patients leading to more than 20% rate of death in these patients; in addition, there is a high prevalence of vitamin D deficiency in these patients; therefore, vitamin D treatment is of high importance in traumatic brain injury (TBI) patients [7–9]. Moreover, vitamin D deficiency is also related to serious complications such as coma, slow neurological recovery, and critical illness polyneuropathy in TBI patients [10, 11]. Although there has been a direct association between vitamin D deficiency and adverse clinical outcomes in critically ill patients, the data regarding vitamin D supplementation from randomized clinical trials are limited [12]. In addition, vitamin D supplementation has not been shown whether it efficiently affects the endpoints of TBI patients or not [4, 9]. The type of vitamin D supplement, route of intervention, and speed of normalization may be associated with clinical outcomes in critically ill patients [4, 9, 12]. As compared to vitamin D injection, enteral vitamin D administration seems to be more effective and is considered safe [13, 14]. To date, studies on the effects of vitamin D on TBI patients have been inadequate and could not provide definitive conclusions [15–17]. The purpose of the present study is to determine the effect of high-dose vitamin D (cholecalciferol) supplementation versus low dose on mortality rate and inflammatory cytokine levels, in critically ill TBI patients under enteral nutrition therapy at intensive care unit (ICU).

## Methods/design

### Study design

**Fig. 1:**
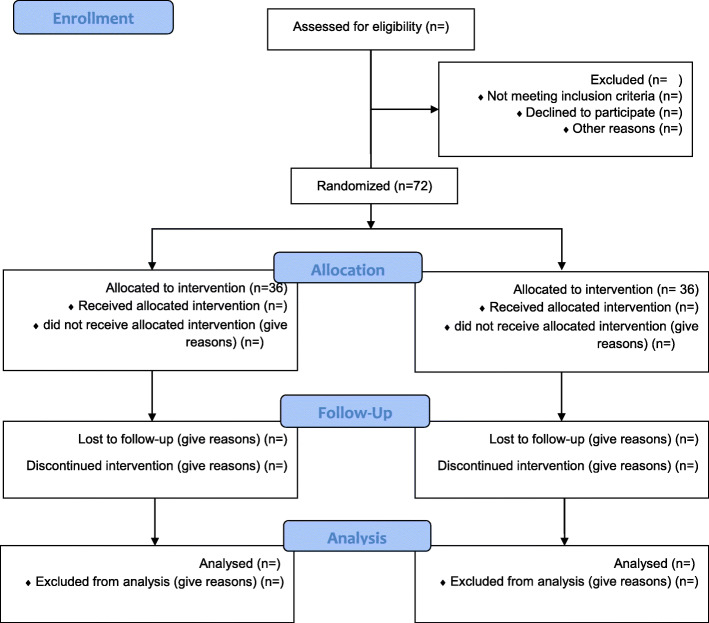
Participant flow diagram according to the Consolidated Standards of Reporting Trials (CONSORT) 2010 statement

**Fig. 2:**
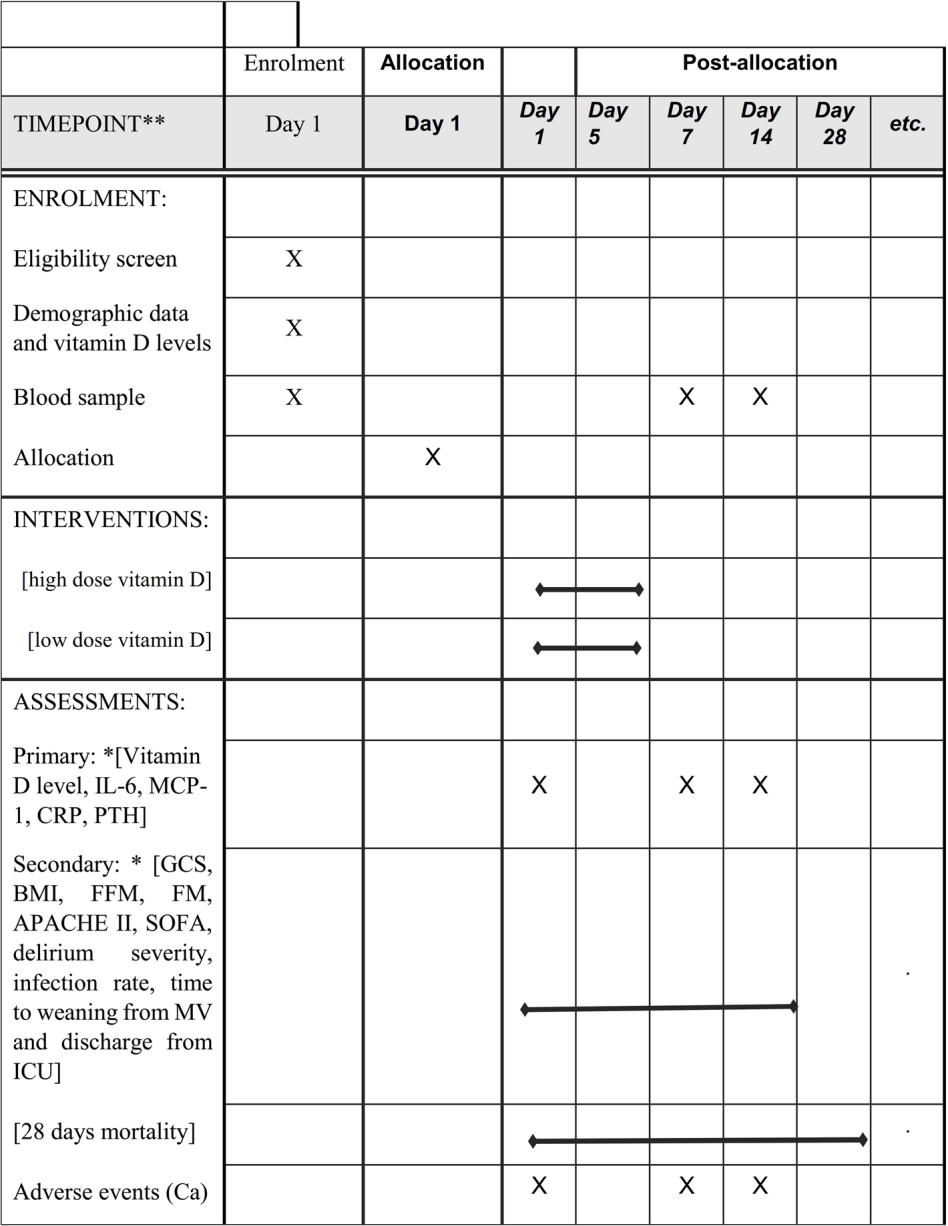
SPIRIT figure showing the schedule of interventions and assessments. IL-6, interlukine-6; MCP-1, monocyte chemoattractant protein-1; CRP, C-reactive protein; PTH, parathyroid hormone; GCS, Glasgow Coma Scale; BMI, body mass index; FFM, fat-free mass; FM, fat mass; APACHE II, Acute Physiology and Chronic Health Evaluation II; SOFA, Sequential Organ Failure Assessment; MV, mechanical ventilator

The treatment of vitamin D deficiency in neurocritically ill patients (VITdAL-ICU) protocol is designed according to the CONSORT guidelines for randomized clinical trial (RCT) [18]. The protocol of this RCT is approved by the Medical Ethics Committee of Mashhad University of Medical Science (IR.MUMS.MEDICAL.REC.1397.381) and is registered at the Iranian Registry of Clinical Trials under IRCT20180619040151N3. The flow diagram illustrates details of the current study protocol in Fig. 1. Moreover, the study time framework for screening, supplementation, and monitoring is described in Fig. 2.

### Study objectives and rationale

The primary goal of the present study is to investigate the effect of daily intake of high-dose vitamin D (cholecalciferol) supplements (100,000 IU) versus low-dose vitamin D supplements (1000 IU) on mortality rate and inflammatory markers through a double-blind, randomized, controlled clinical trial on TBI patients. According to previous observational studies, TBI patients with vitamin D deficiency had higher mortality rate and neuroinflammation in comparison with TBI patients with vitamin D sufficiency [15, 17]. Also, as vitamin D deficiency is highly prevalent in critically ill patients, rapid normalization of this deficiency may be beneficial to these patients [7, 19, 20]. For this reason, we hypothesized that rapid correction of vitamin D deficiency in TBI patients may improve inflammation and decrease mortality rate. This study will also evaluate seven secondary outcomes that may be influenced by vitamin D administration in neurocritical patients. In order to determine the effects of high-dose vitamin D intervention, (1) Glasgow Coma Scale (GCS), (2) time of final weaning, (3) time of discharge from ICU, (4) SOFA (Sequential Organ Failure Assessment) score, (5) APACHE II (Acute Physiology and Chronic Health Evaluation II) score, (6) delirium severity, and (7) parathyroid hormone (PTH) test will be assessed in the treatment group compared to the control group. The study participants will be recruited from adult wards at trauma referral hospitals namely Kamyab and Taleghani in Mashhad, Iran. This study is a phase II RCT, and it has started from August 2019 and will carry on for 5 months.

### Eligibility criteria

Eligibility criteria for TBI patients are listed below. Participants will be allocated into four equal blocks randomized into two groups using an online randomization list.

### Inclusion criteria

1. Patients: traumatic brain injury adults aged 18–65 years old
2. Admission to neurocritical care unit with all type of TBI diagnosis
3. Glasgow Coma scale 7–9
4. TBI patients with vitamin D (25-hydroxy vitamin D3) levels lower than 20 ng/ml
5. Received informed consent from patient’s parents or family members prior to intervention

### Non-entry criteria

1. Patients with hypercalcemia (Ca > 10.8 mg/dl)
2. TBI patients who are NPO more than 48 h or have started total parenteral nutrition (TPN)
3. Severe and active bleeding
4. Patients treated with inotropic and corticosteroid drugs
5. Patients treated with therapeutic dose of any vitamins and minerals apart from the routine ICU protocol
6. Patients with body mass index (BMI) > 40 kg/m^2^ and BMI < 17 kg/m^2^
7. History of any disease such as autoimmune disorders, cancer, sepsis, infection, liver disorder, kidney disorder, diabetes, heart failure, and metabolic diseases
8. Known pregnancy and lactation
9. Patients who are transferred from other ICUs after > 1 week

### Exclusion criteria

- Death before 7 days of TBI patients
- Request to stop the study by patients’ parents or family members
- Patients treated with a different medication protocol
- If items 1 to 5 of non-entry criteria are met

### Study design and setting

We will include 74 TBI patients with epidural hemorrhage (EDH), subdural hemorrhage (SDH), and subarachnoid hemorrhage (SAH) as diagnosed according to computed tomography (CT) scan or magnetic resonance imaging (MRI) findings by the neurosurgeon. Patients will receive vitamin D (cholecalciferol) drop (100,000 IU) or 1000 IU identical control drop in a 1:1 ratio through random assignment method. Vitamin D drops are manufactured by Zahravi Company under good manufacturing practice (GMP). Each vial of vitamin D drop is produced to be dissolved completely in 1 ml of extra virgin olive oil (Familia Company), through a nasogastric tube (NGT) for 5 days in TBI patients. Both high-dose and low-dose drops will have the same bottle, flavor, and aroma to the blinded.

### Sample size

To calculate the sample size, in accordance with a previous study [7], interleukin 6 (IL-6) will be used as the main variable and the type 1 error is considered with an alpha of 0.05. We estimated 62 TBI patients with 80% power to detect an effect size of 0.5 for reduction of IL-6 between the intervention and control groups as calculated with the formula shown below. Considering 20% dropouts in this study, the total sample size is estimated to be 37 participants in each group as calculated in the below formula [7]:

$$ \frac{\left(Z1-\frac{\alpha }{2}+Z1-\beta \right)2\ \left(\delta 12+\delta 22\right)}{\left(\upmu 1-\upmu 2\right)2} $$

### Randomization and blinding

Block randomization method will be considered for this trial study. TBI patients will be randomized (in four blocks) into the intervention group and control group based on a blinded randomization list generated and online databases for clinical trials (https://www.sealedenvelope.com) and will be managed by the research director of Clinical Nutrition Department, Mashhad University of Medical Sciences. Patients will be allocated by random according to the severity of brain injury (GCS 7–8 and 8–9), type of brain injury (EDH, SDH, and SAH), gender, and age to ensure match distribution of these factors in all four blocks. Given that the ICU supervisor has no knowledge of which vial contains high dose or low dose of vitamin D, TBI patients will be randomized to group A or B. The label A or B on vials will be deleted by the research director before allocating the study supplements; thus, the medication will appear similar to the study researcher and nurses. The TBI patient’s study identification code will be documented, and the intervention group may only be determined by comparing the patient’s study number to the reference blinded list, which only the research director will have access to until the trial is finished. Figure 2 indicates the SPIRIT schedule of evaluations and interventions.

### Proposed analysis

To determine the normal distribution of variables, the Kolmogorov-Smirnov test will be conducted. The analysis will be performed according to the intention-to-treat (ITT) test. We perfomed an independent t-test or the Mann-Whitney test for continuous variables and the chi-square or Fisher exact test for categorical variables. Repeated measure one-way analysis of variance (ANOVA) or multivariable covariance analysis (ANCOVA) will be conducted to examine differences in severity of brain injury score (GCS), APACHE II score, SOFA score, delirium score, and all variables at study baseline between the two groups. Kaplan-Meier test was conducted and compared with the use of the log-rank test for estimates of survival time. The *P* values less than 0.05 will be considered statistically significant.

### Primary efficacy endpoint

The primary efficacy outcome for this trial will be assessed by comparing the changes in vitamin D levels and inflammatory markers (IL-6, monocyte chemoattractant protein-1 (MCP-1), and C-reactive protein (CRP)) between two groups. The rate of mortality in TBI patients until 28 days after admission (in ICU and regular ward) will be assessed in high- and low-dose vitamin D supplement groups. The impact of high dose versus low dose of vitamin D will be analyzed, with and without adjustment for age, sex, type of brain injury, and severity of injury. If the results differ after adjusting for the variables, the results of both analyses will be reported.

### Secondary efficacy endpoints

All analyses will be conducted with and without adjustment for age, sex, type of brain injury, and severity of the injury. Time to event analysis will be analyzed similarly as the primary endpoint. The secondary outcomes are listed below:

- Comparison of GCS score changes between the intervention and control groups during ICU
- Comparison of APACHE II and SOFA scores between the intervention and control groups during ICU
- Comparison of incidence of delirium between the intervention and control groups during ICU
- Comparison of changes in PTH levels between the intervention and control groups during ICU
- Comparison of time of discharge from ICU in patients treated with high-dose and low-dose vitamin D groups (during the first 28 days of admission)
- Comparison of time to weaning from mechanical respiratory support in patients treated with high-dose and low-dose vitamin D groups (during the first 28 days of admission)
- Comparison of need for a tracheostomy between the intervention and control groups during ICU stay
- Comparison of occurrence of infections among the intervention and control groups during ICU stay

### Study assessments

**Fig. 3:**
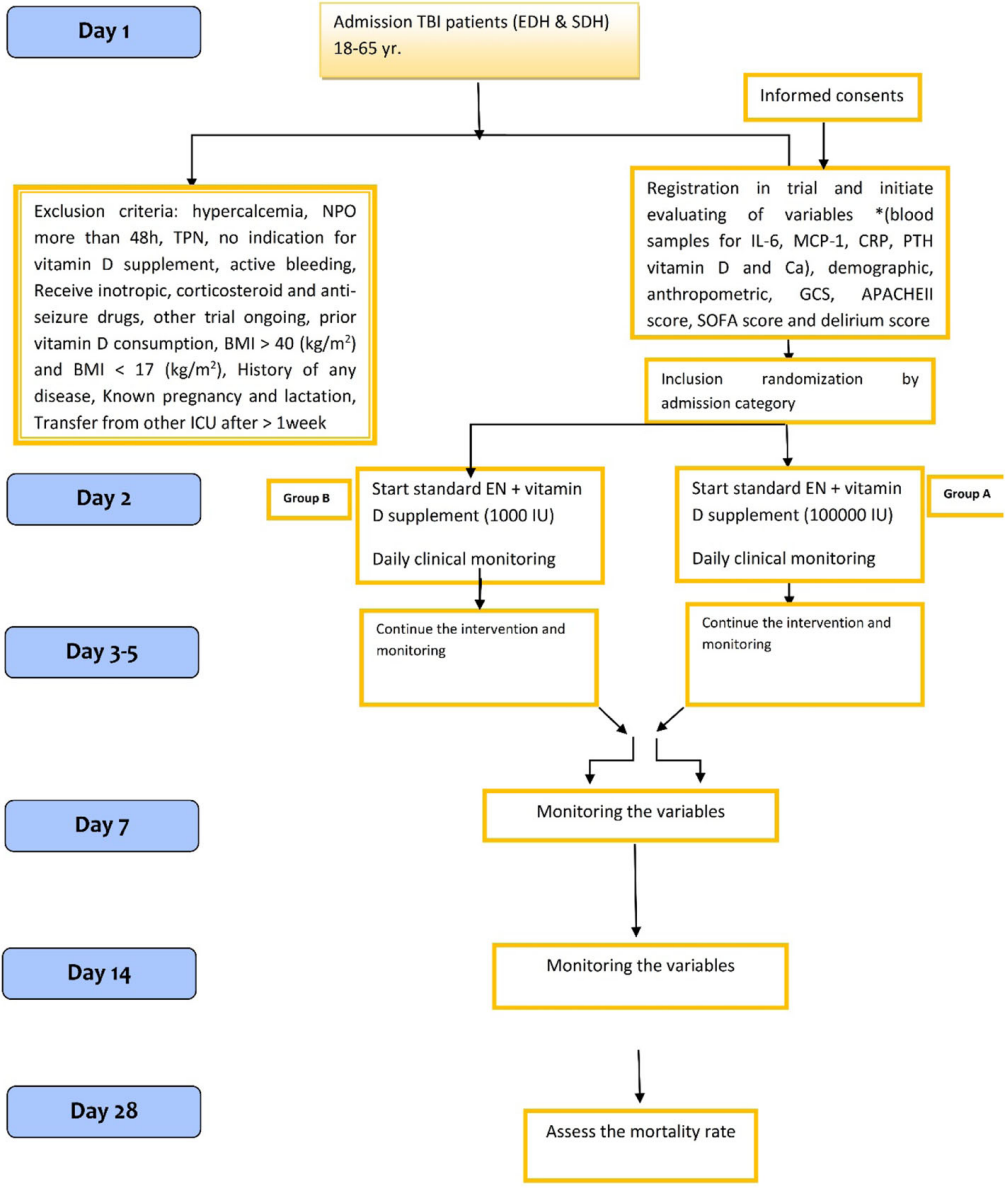
Trial procedure flow sheet

At the beginning of the trial, demographic information, anthropometric parameters (weight (bed scale, Balas Company), estimating height from ulna length), mid arm circumference (measured by tape at the mid-point between the tip of the shoulder and the tip of the elbow), fat-free mass and fat mass (bioelectrical impedance analysis s10, InBody Company), vitamin D3 status (25-hydroxy vitamin D3, chemiluminescence method), IL-6 (ELISA kit), MCP-1 (ELISA kit), quantitative CRP (ELISA kit), PTH (ELISA kit), Ca, APACHE II score, SOFA score, and delirium score will be measured and repeated on days 7 and 14 after intervention in groups A and B. All drugs will be ordered daily upon physician’s request during ICU stay and will be recorded. For adjusting the effect of energy on final outcomes, based on ESPEN guideline for all patients, 25 kcal/kg actual body weight energy is considered [21]. Moreover, the total kilocalories and macronutrient delivered by enteral feeding will be calculated daily for all the participants. Blood samples (5 ml) will be collected at baseline (ICU admission) and 7 and 14 days after intervention at 10:00 am. Blood sample will be taken from veins and centrifuged at room temperature, then stored at − 20 °C. Finally, the samples will immediately be transferred with the cold box to a freezer − 80 °C for future inflammatory cytokine measurements. All routine biochemistry, hematology, and urine tests, until day 14, will be measured after sampling in Kamyab Hospital Lab, Mashhad, Iran. Serum IL-6, MCP-1, and CRP levels will be analyzed in the baseline and days 7 and 14 at the Immunology Lab of Bu Ali Research Institute, Mashhad, Iran. In addition, in case of occurrence of infections (blood stream, the urinary tract, the gastrointestinal tract, wounds, and the lung), an internist specialist will take responsibility [22]. A summary of the schedule of registration, supplementation, and study evaluation is shown in Fig. 3.

### Safety

Vitamin D deficiency treatment is required for patients with low vitamin D levels (< 20 ng/ml) [1]. However, the primary side effect of cholecalciferol supplementation is hypercalcemia [13]. To prevent hypercalcemia in TBI patients, serum vitamin D and calcium levels are evaluated regularly; as adequate vitamin D levels are reached, hypercalcemia is likely to be treated; therefore, the intervention will be stopped [23]. Moreover, to keep track of the patient’s condition, daily vital signs and clinical examinations by neurosurgeon and ICU supervisor will be carried out and will be stopped if the patient’s condition deteriorates.

## Discussion

In the present study, if vitamin D significantly reduces the rate of mortality and inflammation in TBI patients, it can potentially provide the background for larger and well-designed multicenter clinical trial studies to establish the effect of vitamin D supplementation on TBI patients. Furthermore, vitamin D treatment is inexpensive and safe with minimal side effects and also could have positive impacts on critically ill patients (such as decreased mortality rate and hospital length) according to previous studies [7, 13, 14, 23, 24]. Potential limitations of the proposed study are as follows: measurement of vitamin D3 concentration with the chemiluminescence test instead of the gold standard method (liquid chromatography-tandem mass spectrometry). Another limitation is TBI patients’ blood transfusion because of trauma and bleeding; some TBI patients may require blood transfusion during the study, which may affect the biochemistry outcomes. Albumin injection in hypoalbuminemia patients can affect the levels of serum albumin. Also, TBI patients may need to undergo other surgeries than brain surgery; thus, this factor could affect the study outcomes. For this reason, we will compare mortality rate and inflammatory cytokine levels in all participants (intervention and control) and separate patients with anemia and hypoalbuminemia in each group. If the rate of death or inflammation in patients with anemia and hypoalbuminemia is different from those in the case or control group, the results will be reported separately. Based on recent studies, we have considered the suggested vitamin D dose (100,000 IU/day for 5 days) by Han et al. [23], as it is shown to be safe and efficacious in critically ill patients. Therefore, we believe that the present study could elaborate the necessity of vitamin D treatment and its efficacy on TBI patients at ICU.

## Conclusion

The present clinical trial has begun in June 2019 and will be completed in November 2019. If comprehensive results are observed in the clinical outcomes of TBI patients, vitamin D treatment could be conducted as a new approach for optimizing the TBI patient care at ICU.

### Trial status

This trial (protocol version number version 1.1, November 13, 2017) is ongoing. Participant recruitment began on August 11, 2019, and completed on December 21, 2019. The trial procedures are expected to be completed by the end of January 2020.

## Data Availability

Final data for the present clinical trial can be made accessible upon email request. Interested researchers should contact Dr. Abdolreza Norouzy at Norouzya97@gmail.com.

## Abbreviations

VITdAL-ICU: The correction vitamin D deficiency in critically ill patients
ICU: Intensive care unit
TBI: Traumatic brain injury
EDH: Epidural hemorrhage
SDH: Subdural hemorrhage
SAH: Subarachnoid hemorrhage
EN: Enteral
TPN: Total parenteral nutrition
BMI: Body mass index
NPO: Nothing by mouth
RCT: Randomized controlled trial
GCS: Glasgow Coma Scale
APACHE II: Acute Physiology and Chronic Health Evaluation II
SOFA: Sequential Organ Failure Assessment
IL-6: Interleukin 6
MCP-1: Monocyte chemoattractant protein-1
CRP: C-reactive protein
PTH: Parathyroid hormone
Ca: Calcium
LOS: Length of stay
